# Exploration on the Mechanism of Ubiquitin Proteasome System in Cerebral Stroke

**DOI:** 10.3389/fnagi.2022.814463

**Published:** 2022-04-07

**Authors:** Yu-Chao Li, Yan Wang, Wei Zou

**Affiliations:** ^1^Heilongjiang University of Chinese Medicine, Harbin, China; ^2^School of Traditional Chinese Medicine, Ningxia Medical University, Yinchuan, China; ^3^First Affiliated Hospital, Heilongjiang University of Chinese Medicine, Harbin, China

**Keywords:** UPS, stroke, ubiquitination, mitochondrial dysfunction, oxidative stress, inflammation

## Abstract

Stroke’s secondary damage, such as inflammation, oxidative stress, and mitochondrial dysfunction, are thought to be crucial factors in the disease’s progression. Despite the fact that there are numerous treatments for secondary damage following stroke, such as antiplatelet therapy, anticoagulant therapy, surgery, and so on, the results are disappointing and the side effects are numerous. It is critical to develop novel and effective strategies for improving patient prognosis. The ubiquitin proteasome system (UPS) is the hub for the processing and metabolism of a wide range of functional regulatory proteins in cells. It is critical for the maintenance of cell homeostasis. With the advancement of UPS research in recent years, it has been discovered that UPS is engaged in a variety of physiological and pathological processes in the human body. UPS is expected to play a role in the onset and progression of stroke via multiple targets and pathways. This paper explores the method by which UPS participates in the linked pathogenic process following stroke, in order to give a theoretical foundation for further research into UPS and stroke treatment.

## Introduction

Stroke is a potentially fatal cerebrovascular event defined by brain tissue damage produced by a sudden rupture of cerebral vessels or cessation of cerebral blood supply, resulting in neurological dysfunction, including ischemic and hemorrhagic stroke (Landowski et al., 2020). Stroke has overtaken ischemic heart disease as the second largest cause of death worldwide (Benjamin et al., 2017; Li et al., 2021). According to a comprehensive analysis of Chinese population’s health, stroke has become the leading cause of mortality in the country (Zhou et al., 2019). According to current research, the ubiquitin proteasome system (UPS) plays a role in the molecular processes that lead to the occurrence and progression of stroke. UPS is also linked to a number of signaling pathways that cause injuries after stroke.

## Ubiquitin Proteasome System

In cells, UPS is the primary non-lysosomal route for protein breakdown. This system destroys proteins with basic functions in addition to misfolded or oxidized proteins. Under physiological and pathological situations, it is a critical mechanism for maintaining protein homeostasis (Kramer et al., 2021). In addition, the system is participated in a variety of cellular functions, including DNA repair (Uckelmann and Sixma, 2017), endocytic trafficking (Hicke, 2001), and immunological response (Bednash and Mallampalli, 2016). UPS is vital in central nervous system disorders because it can clean up aberrant proteins in neurodegenerative diseases including Alzheimer’s and Parkinson’s disease (Graham and Liu, 2017). At the same time, the system controls the primary risk factors for cerebrovascular disease, such as atherosclerosis (Wilck and Ludwig, 2014), hypertension (Li et al., 2013), hyperlipidemia (Sharpe et al., 2020), type 2 diabetes (Sun-Wang et al., 2021), and so on.

### Ubiquitination Process

Ubiquitination is usually mediated by UPS. Ubiquitin, ubiquitin-activating enzyme (E1), ubiquitin-conjugating enzyme (E2), ubiquitin protein ligase (E3), deubiquitinase (DUB), and the proteasome are all important components of the UPS (Kane et al., 2021). Ubiquitin is a tiny peptide with a molecular weight of about 8.5 kDa that consists of 76 amino acids. It has a highly conserved sequence and is found in eukaryotic cells in large quantities. Ubiquitin molecule contains seven lysine residues (K6, K11, K27, K29, K33, K48, K63) and an N-terminal methionine residue (M1), which allow ubiquitin molecules to connect to one another (Kwon and Ciechanover, 2017). Homotypic polyubiquitination modification and heterotypic polyubiquitination modification are two types of ubiquitin-ubiquitination modification (Komander and Rape, 2012). Furthermore, ubiquitin can undergo post-translational modifications such as phosphorylation or acetylation (Yau and Rape, 2016; Ohtake and Tsuchiya, 2017). This complicates the ubiquitination process. Generally, E1 forms a high-energy thiohydroxyester bond between the carboxyl group of ubiquitin’s C-terminal glycine and the sulfhydryl side chain of E1’s active cysteine under the action of ATP (Ciechanover, 2015). E2 is an intermediate enzyme in ubiquitination reaction, which can bind E1 and E3. E2 enzyme has a highly conserved ubiquitin-conjugating enzyme domain (UBC). On the UBC sequence, there is a cysteine site with symbolic activity, which can accept ubiquitin molecules activated by E1 and form a thioester bond with ubiquitin (Zheng and Shabek, 2017). Subsequently, E2 with activated ubiquitin binds to E3; Finally, E2 transfers the ubiquitin molecule to the substrate by inducing the formation of an isopeptide bond between the C-terminus of ubiquitin and a target lysine of the substrate. E3 specifically recognizes target proteins during ubiquitination (Buetow and Huang, 2016).

E3’s function reflects selectivity and efficiency of ubiquitination. Single subunit proteins and multisubunit complexes are two types of E3. There are four families of E3 single subunit proteins that have been identified: the HECT domain family, the RING domain family, the U-box domain family, and the N-recognition family. Cullin-RING and APC/C are the most common multisubunit complexes found in E3 (Zheng and Shabek, 2017). The human genome encodes approximately more than 600 ubiquitin ligases E3. Different E3 enzymes can specifically mediate the formation of different ubiquitin chain types. According to the different types of ubiquitin chain modification, it shows different functions. Polyubiquitination modification mediated by K48 or K11 ubiquitin chain is involved in proteasome degradation. Many E3 enzymes, such as SCF, gp78 and E6AP, can form K48 ubiquitin chains on substrate proteins, thus mediating the degradation of substrate proteins through proteasome (Wang and Pickart, 2005; Gao et al., 2016; Thacker et al., 2020). K11 ubiquitin chain was first considered as a regulator of cell fission, and its abundance increased with the increase of APC/C activity. APC/C, as an E3 enzyme, mediates the substrate Cyclin B1 to form K11 ubiquitin chain, which is finally degraded by proteasome, so as to promote the normal progress of cell mitosis (Song and Rape, 2010). In addition, studies have shown that K11 ubiquitin chain is often difficult to bind to 26S proteasome, but its degradation efficiency will be significantly enhanced when it forms a composite chain with K48 (Grice et al., 2015). Previous studies have shown that linear ubiquitination assembly complex (LUBAC) can modify NEMO by M1 ubiquitin chain to activate NF-κB pathway (Rahighi et al., 2009). This indicates that M1 ubiquitin chain is involved in activation of NF-κB pathway. ITCH and SMURF1 of the HECT family are members of the K29 ubiquitin chain’s ubiquitin ligases. SMURF1 has been found to add K29 ubiquitin chain to AXIN in WNT pathway. AXIN modified by K29 ubiquitin chain will not be degraded, but will lose the ability to interact with receptor LRP5/6 of WNT pathway (Fei et al., 2013). ITCH has been reported to catalyze Deltex in the NOTCH pathway to form K29 ubiquitin chain, which enables Deltex to enter the lysosomal pathway (Chastagner et al., 2006). In the process of DNA damage repair, RNF168 will form K27 polyubiquitin chain on histone, and then recruit 53BP1 and BRCA1 to DNA damage site to start the DNA damage repair (Brown and Jackson, 2015; Gatti et al., 2015).

### Proteasome

**FIGURE 1:**
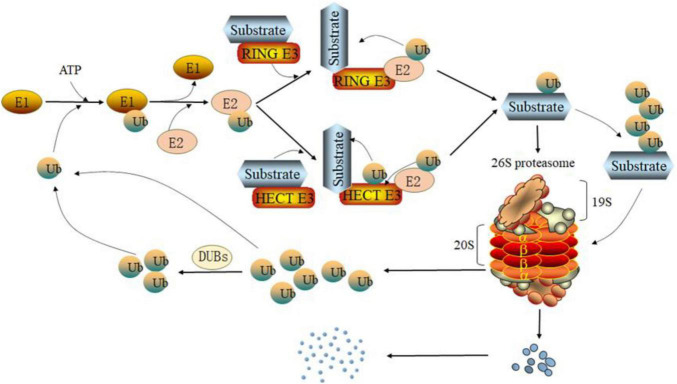
Degradation pathway of the ubiquitin proteasome system (UPS). Under the action of ATP, the ubiquitin activating enzyme E1 forms a high-energy thiohydroxyester bond between the carboxyl group of ubiquitin’s C-terminal glycine and the sulfhydryl side chain of E1’s active cysteine. Then, ubiquitin-conjugating enzyme E2 receives ubiquitin activated by E1 through a cystine site on its ubiquitin-conjugating enzyme domain (UBC) and forms a thioester bond. Depending on the type of ubiquitin protein ligase E3, E2 can transfer ubiquitin to the substrate directly (RING E3s) or indirectly (HECT E3s) and form an isopeptide between the substrate and ubiquitin. In addition, ubiquitin can itself be the target of the ubiquitination cascade. Finally, the ubiquitinated substrate is degraded by the 26S proteasome. The ubiquitin separated from the substrate will re-enter the ubiquitination cycle.

The proteasome is a multi-subunit protease complex that is found in both the cytoplasm and the nucleus. In mammalian cells, it is the primary neutral protein hydrolase. It is ubiquitin-dependent and possesses a variety of protein hydrolase functions. In UPS, the term “proteasome” usually refers to the 26S proteasome. The 20S proteasome and the 19S subcomplex make up the 26S proteasome (Gillette et al., 2008). Before the target proteins enter the 20S proteasome, each 19S sub complex comprises several ATPase active sites and ubiquitin binding sites that can recognize ubiquitinated target proteins, deubiquitinate the target proteins, and open the structural folding of the target proteins (Matyskiela et al., 2013). The 20S proteasome of eukaryotic cells contains two α rings and two β rings, these α rings and β rings together form a cylindrical structure, both α rings or β rings are composed of seven subunits. α rings are found in the outer layer of the 20S protease and are mostly employed for substrate identification; β rings are found in the inner layer and are primarily responsible for substrate degradation (Fricker, 2020). The β1, β2, and β5 of the seven β subunits have proteolytic activities, which are referred to as caspase-like activity, trypsin-like activity, and chymotrypsin-like activity, respectively, enzymolysis of acidic, alkaline, hydrophobic, or aromatic amino acid residues at the carboxyl end (Unno et al., 2002; Figure 1).

## Occurrence and Development of Stroke

According to the causes, stroke can be classified as hemorrhagic or ischemic, with ischemic stroke accounting for around 87% of all cases (Virani et al., 2021). Due to cerebral ischemia or hemorrhage, the normal blood supply of neurons is destroyed, which leads to a series of pathophysiological reactions and finally to the death of nerve cells. The mechanisms involved in nerve injury include excitotoxicity, mitochondrial dysfunction, free radical disorder, inflammation, apoptosis, necrosis, autophagy and so on. According to analyses based on data from the Global Burden of Disease (GBD) study, modifiable risk factors such as hypertension, obesity, hyperglycemia, hyperlipidemia, atrial fibrillation and renal dysfunction account for 87% of stroke risk, while behavioral risk factors such as smoking, sedentary behavior, and an unhealthy diet account for 47%. Air pollution was found to be responsible for 30% of the global risk of stroke (Collaborators, 2020). In addition, age, gender and race are also associated with stroke (Diseases and Injuries, 2020). The combined action of multiple risk factors leads to pathological changes of cardiovascular and cerebrovascular system, including but not limited to atherosclerosis, arteriolar fat hyalinization and fibrin like necrosis, coronary artery disease, and myocardial injury. These pathological changes provide clues for the occurrence, recurrence, and secondary prevention of stroke.

Although brain tissue makes up only 2% of total body weight, cerebral blood flow accounts for 15% of cardiac output. The aerobic oxidation of glucose provides the majority of the energy required by brain tissue, although the glucose reserve is limited. Though brain oxygen use contributes for 23% of total body oxygen consumption, the oxygen reserve is small (Hyder et al., 2013). For these factors, the brain is extremely vulnerable to ischemia and hypoxia. Following a stroke, brain ischemia, and hypoxia are major concerns. In the ischemic core the major mechanism of cell death is energy failure. Neurons cannot create the ATP needed to supply the ionic pumps that maintain the ionic gradient across the neuronal membrane, primarily the Na^+^/K^+^ ATPase, without oxygen or glucose. As a result, a substantial amount of Na^+^ and Ca^2+^ accumulate in the cytoplasm, causing organelles swelling and degeneration, loss of cell membrane integrity, and ultimately cell necrosis (Lipton, 1999). In the ischemic penumbra, the decrease in blood flow due to collateral blood supply is not enough to lead to rapid energy failure, and neurons still survive for a long time after ischemia and hypoxia. The excessive accumulation of extracellular glutamate is an important factor leading to ischemic penumbra injury (Mazzocchetti et al., 2020). Due to the excessive accumulation of glutamate, the overactivation of subtypes of N-methyl-D-aspartate receptors (NMDARs) leads to cellular calcium overload. Therefore, calcium-dependent proteases are activated, such as calpains, resulting in nerve cell damage (Rami et al., 2000). In addition, some studies have shown that calpains are involved in the activation of Caspase-3, which may be an important mechanism of neuronal apoptosis after stroke (Blomgren et al., 2001). Meanwhile, mitochondrial dysfunction is caused by Ca^2+^ overload which leads to mitochondrial permeability transition pore (mPTP) opening (Zhu et al., 2018). Of note, mitochondrial dysfunction leads to insufficient ATP production, which will further lead to Ca^2+^ accumulation and form a vicious circle. Nerve cell survival is aided by the removal of defective mitochondria. Mitochondrial autophagy is an important regulatory process for the quality and quantity of mitochondria. According to a report, UPS plays a key function in the regulation of mitochondrial autophagy (Alsayyah et al., 2020). Mitochondria are organelles that operate as oxidative energy centers and are required for cell survival, but aging or damaged mitochondria produce deadly reactive oxygen species (ROS; Liu et al., 2018). ROS are important signaling molecules in oxidative stress. In the pathological manifestations of stroke, relatively excessive ROS will destroy the homeostasis of intracellular environment, resulting in oxidative stress and mitochondrial damage (Sims and Muyderman, 2010). The main defensive response to oxidative and electrophilic stressors is the Keap1-Nrf2 pathway. UPS can strictly regulate the transcription of nuclear factor erythroid2-related factor (Nrf2) through this pathway, thereby affecting the antioxidant process (Baird and Yamamoto, 2020). Whether it is mitochondrial dysfunction or oxidative stress, it will eventually lead to neuronal damage. Inflammation is one of the most prevalent pathological signs following a stroke, and it is induced by a range of factors, including microglia activation (Koh et al., 2018) and cytokine involvement (Xu et al., 2018). Inflammation has the potential to be neurotoxic., which can lead to neuronal death (Liddelow et al., 2017). Hypoxia-inducible factor-1 (HIF-1) comprised by α and β subunits is a protein that regulates the expression of genes that code for erythropoietin (EPO) and vascular endothelial growth factor (VEGF), as well as genes involved in glucose transport and glycolysis, such as glucose transporter-1 (GLUT1), pyruvate dehydrogenase kinase 1 (PDK1), and lactate dehydrogenase A (LDHA; Leu et al., 2019). In the hypoxic state caused by stroke, the oxygen-dependent HIF prolyl hydroxylase domain (PHD) is inactivated, leading to the stabilization of HIF-1α, followed by its translocation into the nucleus, where it forms a heterodimeric complex with HIF-1β. This complex, called HIF-1, interacts with DNA and activates the expression of multiple target genes encoding proteins that help increase the tissue’s oxygen supply by boosting erythropoiesis and angiogenesis (Semenza, 2004, 2009). UPS has been implicated in the control of HIF in recent research (Delpeso et al., 2003). Therefore, regulating related pathways and their key proteins through UPS may be an effective solution to protect neurons and prevent cell death during stroke.

## Regulatory Mechanism of Ubiquitin Proteasome System

### NF-κB Pathway and Ubiquitin Proteasome System

NF-κB is a transcription factor family that includes NF-κB1 (P50/p105), NF-κB2 (p52/P100), and three RelA (p65), RelB, and c-Rel (REL) proteins (Caamano and Hunter, 2002). As a hub in signal transduction pathway, NF-κB can regulate the expression of many genes involved in cell inflammation, immune response, cell growth and development (DiDonato et al., 2012). IκB protein is a family of constitutive inhibitors of NF-κB, including IκBα, IκBβ, IκBγ, IκBζ, IκBε, Bcl-3, p100 and p105 (Yamazaki et al., 2001; Chiba et al., 2013). IKK complex consists of IKKα, IKKβ and Nemo, which is the kinase of IκB (Durand and Baldwin, 2017). Under normal conditions, NF-κB and inhibitor IκB binding exists in the cytoplasm in an inactive potential state (Wang et al., 2019).

**FIGURE 2:**
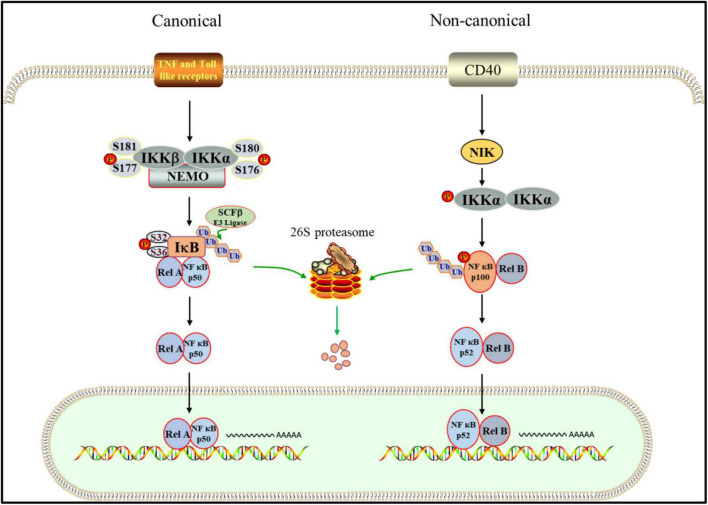
UPS is involved in canonical and non-canonical NF-κB pathway. In the canonical way. After the inflammatory receptor is activated, the activated IKK complex phosphorylates ser32 and ser36 of IκB, which promotes the ubiquitination of IκB mediated by SCFβ. Subsequently, IκB is targeted to the 26S proteasome for proteolysis. NF- κB dimer was released and transferred into the nucleus to start the transcription of target genes. In the non-canonical pattern. IKK is activated by NIK, which leads to phosphorylation of p100. Then, Phosphorylated p100 is degraded by proteasome after ubiquitination, in which p52-RelB heterodimer is produced. Finally, the p52-RelB heterodimer enters the nucleus to start transcription.

NF-κB signaling pathway mainly includes classical and non-classical activation pathways (Figure 2). In the classical one, IKK complex is activated when cells are stimulated by proinflammatory factors, growth factors, immune receptor ligands and stress response (Fricker, 2020), in which IKKβ (ser177 and ser181) and IKKα (ser176 and ser180) sites in the amino terminal kinase domain are phosphorylated. Due to their activation, serine residues of IκB are phosphorylated (Ling et al., 1998; Delhase et al., 1999). The polyubiquitination mode of IκBα depends on the phosphorylation of two serine residues (ser32 and ser36), while the phosphorylation sites of IκBβ are ser19 and ser23 (Chen, 2012). The phosphorylation of IκB leads to the covalent binding of lysine residues at positions 21 and 22 of its amino terminal to multiple ubiquitin molecules through SCF type E3 ubiquitin ligase complex. This binding changes the spatial conformation of IκB, resulting in its recognition and degradation by ATP dependent 26S proteasome (Chen et al., 1995; Karin and Ben-Neriah, 2000; Caamano and Hunter, 2002). Following depolymerization, the liberated NF-κB dimer is activated by several post-translational modifications before being transported to the nucleus and combining with particular DNA sequences to increase target gene transcription (Zhang et al., 2017). Inflammatory cytokines like TNF-α, IL-2, IL-6, and INF-γ are then produced, reactivating NF-κB and causing inflammatory damage to cell tissues (Mitchell and Carmody, 2018). The activation of non-classical NF-κB pathway depends on receptor activated kinase NIK, which can activate IKKα (Cildir et al., 2016). The phosphorylation of NF-κB2 p100 mediated by activated IKKα leads to its own ubiquitination, which is recognized by proteasome and partially degraded to p52. Finally, p52-RelB heterodimer enters the nucleus to start transcription (Xiao et al., 2001; Sun, 2011). Therefore, NF-κB plays a crucial role in acute and chronic inflammatory illnesses as the center regulating the expression of pro-inflammatory genes in the inflammatory process (Baker et al., 2011).

### PINK1/Parkin Pathway and Ubiquitin Proteasome System

**FIGURE 3:**
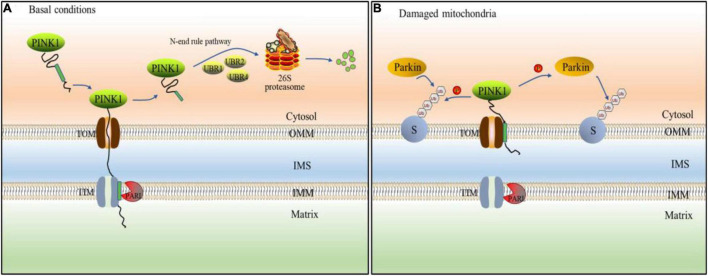
PINK1/Parkin pathway mediates ubiquitination of mitochondria. **(A)** Under basal condition, PINK1 passes through the outer mitochondrial membrane (OMM) to the inner membrane (IMM), and its transmembrane segment is hydrolyzed by presenilins-associated rhomboid-like protein (PARL) protein. The treated PINK1 is released into the cell matrix and mediated proteasome degradation by E3 ubiquitin ligases (UBR1, UBR2, and UBR4). **(B)** When mitochondrial disorder, PINK1 cannot be transported across membranes, so that it cannot be destroyed by PARL. Instead, PINK1 stably binds to the OMM and accumulates on it. Therefore, PINK1 can directly phosphorylate ubiquitin or Parkin. Parkin is recruited by phosphorylated ubiquitin to various mitochondrial substrate proteins(S) in which mitochondrion is ubiquitinated.

The PTEN-induced putative kinase 1(PINK1)/Parkin pathway is one of the most prevalent mechanisms for controlling mitochondrial quality and abundance (Shen et al., 2021). PINK1 is a mitochondrial serine (Ser)/threonine (Thr) kinase with a mitochondrial targeting sequence (MTS), a transmembrane (TM) segment, and a Ser/Thr kinase domain that is nuclear encoded (Eiyama and Okamoto, 2015). PINK1, as a Parkin upstream component, is required for Parkin activation and recruitment to depolarized mitochondria (Miller and Muqit, 2019). Parkin has an N-terminal ubiquitin-like (UBL) domain, three RING domains (RING0, RING1, and RING2), and an in-between RING(IBR) domain that separates RING1 and RING2 as an E3 ligase (Biswas et al., 2020). When the MTS and TM of PINK1 reach the inner mitochondrial membrane under healthy conditions, the transmembrane segment is cleaved in the form of protein hydrolysis by the presenilins-associated rhomboid-like protein (PARL) found in the inner membrane. Cleaved PINK1 is released into the cytoplasm, exposing destabilizing amino acid residues at its N-terminus. E3 ubiquitin ligases (UBR1, UBR2, and UBR4) ubiquitinate them with N-terminal rules and degrade fast by proteasome (Yamano and Youle, 2013; Figure 3A). Therefore, the content of PINK1 in cytoplasm is very low and Parkin is not activated (Pickrell and Youle, 2015). When the mitochondrial membrane potential is abnormal, PINK1 avoids PARL-mediated processing and N-end rule-dependent degradation by forming a stable association with the translocase of the outer membrane (TOM) and accumulating on the outer mitochondrial membrane (OMM; Lazarou et al., 2012). PINK1 accumulates on the OMM can activate parkin in two ways. On the one hand, Parkin is activated by PINK1 phosphorylating ser65 in the Parkin UBL domain (Bingol and Sheng, 2016). On the other hand, PINK1 phosphorylates ser65 in ubiquitin, which is coupled with OMM proteins at basic levels. Parkin’s affinity for phosphorylated ubiquitin is what causes it to be found in mitochondria. The activated Parkin further binds ubiquitin to the OMM protein, and then ubiquitin is phosphorylated by PINK1 (Bragoszewski et al., 2017; Figure 3B). On mitochondria, phospho-ubiquitin produced by PINK1 serves as an autophagy signal, which Parkin amplifies (Lazarou et al., 2015). Subsequently, ubiquitinated OMM protein recruits autophagic adaptor protein SQSTM1/p62 to damaged mitochondria (Geisler et al., 2010), and promotes its degradation through autophagy. There are some negative regulatory mechanisms related to UPS in PINK1/Parkin pathway, which are very important for the stability of mitochondrial autophagy. Ubquitin-specific protease30 (USP30) is an OMM localized DUB, which antagonizes PINK1/Parkin mediated mitochondrial autophagy by deubiquitination of OMM proteins. The presence of USP30 can maintain the steady state of ubiquitination of OMM protein and prevent excessive mitochondrial autophagy (Tanaka, 2020). Furthermore, UBL exhibits a strong affinity for the Rpn13 subunit of the 26S proteasome regulatory granules. The proteasome is attracted to mitochondria by this affinity, which increases proteasome degradation of ubiquitinated OMM protein and Parkin (Aguileta et al., 2015).

### Keap1-Nrf2 Pathway and Ubiquitin Proteasome System

**FIGURE 4:**
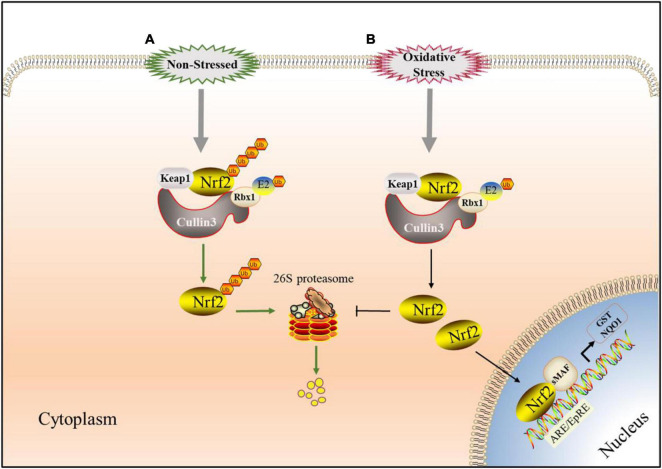
Keap1-Nrf2 signaling pathway involved in UPS. **(A)** Under non-stressed conditions, Nrf2 binds to Keap1 complex which has E3 ligase activity in the cytoplasm. When Nrf2 is ubiquitinated, it is targeted to the 26S proteasome for proteolysis which keep it low in cytoplasm. **(B)** Under oxidative stress conditions, Critical cysteine residues in Keap1 is covalently-modified by electrophilic species or reactive oxygen species (ROS) and Nrf2 avoides Keap1-mediated ubiquitination modification. Then Nrf2 translocated to the nucleus, starts ARE/EpRE transcription through heterodimerization with SMAF protein, and induced the expression of a series of cytoprotective genes.

Nrf2 is the main regulator of redox and metabolic homeostasis. It has seven Nrf2 ECH homologous domains (Neh1-Neh7), and each domain has different functions (Hayes and Dinkova-Kostova, 2014). As the most important regulatory domain of Nrf2, Neh2 includes two motifs, DLG and ETGE, which can regulate the stability and ubiquitination of Nrf2 by binding to other proteins such as Keap1 (Tong et al., 2006). Keap1 is a substrate adaptor protein of Cullin3 (Cul3)-dependent E3 ubiquitin ligase complex, which can be assembled with Cul3 and Rbx1 (ring-box1). Keap1 serves as a substrate adapter, whereas Rbx1 binds to ubiquitin-loaded E2 and Cul3 serves as a scaffold between Keap1 and Rbx1, which can regulate Nrf2 (Tonelli et al., 2018). Keap1 contains three functional domains, including a BTB domain, an IVR and a Kelch or DGR domain (Sajadimajd and Khazaei, 2018). The N-terminal BTB domain of Keap1 can bind Cul3, which is necessary for Keap1 dimerization (Suzuki and Yamamoto, 2015). Under normal conditions, the Neh2 domain of Nrf2 interacts with the Kelch/DGR domain in Keap1 through the mediation of DLG and ETGE motifs. Keap1-Cul3-E3 ubiquitin ligase targets multiple lysine residues located in the Neh2 domain at the N-terminal of Nrf2 and promotes the ubiquitination of Nrf2. The ubiquitinated Nrf2 is transported to the 26S proteasome, where it is degraded (Canning et al., 2015; Karunatilleke et al., 2021; Figure 4A). Critical cysteine residues in Keap1, particularly Cys151, operate as sensors of these cellular damages under oxidative stress conditions and become covalently changed by electrophilic molecules or ROS (Zhang and Hannink, 2003). Such changes cause Keap1 to shift conformation, most likely by disrupting the low-affinity link between the Kelch domain and the DLG-motif, resulting in decreased ubiquitylation of Nrf2, inhibiting UPS-mediated degradation and thereby boosting Nrf2 protein levels (Baird et al., 2013). Then Nrf2 translocases to the nucleus and binds to the ARE/EpRE of the target gene through heterodimerization with sMAF protein to induce the expression of a series of cell protective genes, such as NQO1, GST, HMOX1, GCL, GSH, etc. (Kansanen et al., 2013; Fao et al., 2019; Figure 4B), so as to reduce or eliminate oxygen free radicals and improve the antioxidant capacity of cells and tissues.

### HIF-1 Pathway and Ubiquitin Proteasome System

**FIGURE 5:**
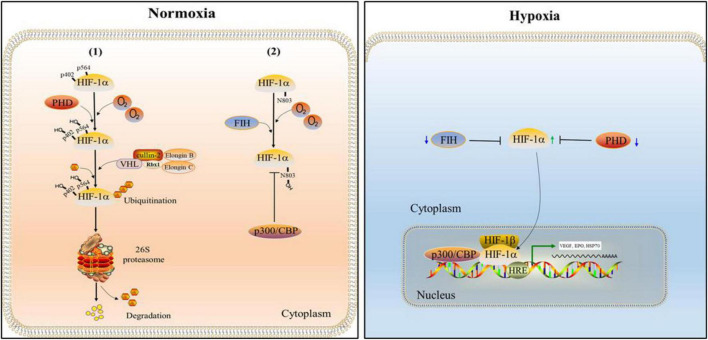
The regulation of HIF-1. Under the normal oxygen condition, the prolyl residues at sites 402 and 564 of HIF-1α are hydroxylated by the prolyl hydroxylase domain (PHD). Subsequently, hydroxylated HIF-1α is ubiquitinated by Von Hippel-Lindau (VHL) and degraded by 26S proteasome. In addition, the asparagine residue (N803) of HIF-1α is hydroxylated by the factor inhibiting HIF (FIH) which inhibits the connection between p300/CBP and C-TAD, thereby reducing the transcriptional activity of HIF-1α. When hypoxia occurs, the activities of FIH and PHD are inhibited. Therefore, HIF-1α can enter the nucleus to form complexes with HIF-1β and p300/CBP, and promote the transcription of hypoxia response genes and cause a series of adaptive responses of cells to hypoxia.

Hypoxia inducible factor (HIF) is an important transcription factor regulated by the change of oxygen concentration (Wang et al., 1995). It is composed of a α subunit regulated by oxygen concentration and a constitutively expressed β subunit, in which α subunit has three functional forms: HIF-1α, HIF-2α and HIF-3α. HIF is divided into HIF-1, HIF-2 and HIF-3 according to different α subunits. α subunit and β subunit have two important domains: the basic helix-loop-helix (bHLH) and Per-Arnt-Sim (PAS). The bHLH and PAS are required for dimerization between HIF-1α and HIF-1β (Zhang et al., 2011; Shu et al., 2019). HIF-1α is generally expressed in all cells, while HIF-2α and HIF-3α are selectively expressed in some tissues (Majmundar et al., 2010). HIF-1α which has two transactivation domains, N-terminal transactivation domains (N-TAD) and C-terminal transactivation domains (C-TAD), as well as an oxygen-dependent degradation domain (ODDD) that mediates oxygen-regulated stability, is not only the regulatory subunit of HIF-1, but also its active subunit (Ruas et al., 2002). The stability and activity of α subunit are regulated by post-translational modifications such as hydroxylation, ubiquitination, acetylation and phosphorylation. Among them, HIF-1α is mainly regulated by the PHD (Ke and Costa, 2006). Under the normoxic state, the prolyl residues at sites 402 and 564 of HIF-1α are hydroxylated by PHD. Hydroxylated HIF-1α binds to Von Hippel-Lindau (VHL), which together with Elongin C, Elongin B, Cullin-2, and Rbx1, forms the VCB-Cul2 E3 ligase complex. Subsequently, it is ubiquitinated, then recognized by 26S proteasome and degraded (Ohh et al., 2000). Since PHD activity is suppressed during hypoxia, VHL is unable to detect HIF-1α, and hence HIF-1α is not degraded by UPS. Subsequently, accumulated HIF-1α enters the nucleus from the cytoplasm, where it is joined to create a dimer with HIF-1β (Tarhonskaya et al., 2015). In the nucleus, p300/CBP associated factor (PCAF) combines with the C-TAD of HIF-1α to form a complex. The complex combines with the hypoxia response element (HRE) in the promoter region of hypoxia response genes to promote the transcription of hypoxia response gene and cause a series of adaptive responses of cells to hypoxia (Dengler et al., 2014). In addition, HIF-1α is also regulated by the factor inhibiting HIF (FIH; Kaelin and Ratcliffe, 2008). In normoxic environment, FIH hydroxylates the asparagine residue (N803) of HIF-1α to prevent the connection between p300/CBP and C-TAD, thereby reducing the transcriptional activity of HIF-1α (Lando et al., 2002b). Under hypoxia, the hydroxylation of FIH is inhibited, which promotes the interaction between HIF-1α and p300/CBP and leads to the transcription of target genes (Lando et al., 2002a; Figure 5).

## The Role of UPS in Signal Pathway After Stroke

### UPS Participation in NF-κB Pathway and Stroke

UPS is essential for maintaining protein homeostasis and preventing damaging protein aggregation in cells (Budenholzer et al., 2017). Inflammation is the result of a complicated interplay between soluble substances and cells (Medzhitov, 2008). Inflammatory cell infiltration and activity frequently result in long-term tissue damage (Feehan and Gilroy, 2019). UPS can play an important role in the inflammatory process by regulating a variety of inflammatory regulatory proteins (Goetzke et al., 2021). It was found that inflammatory response widely exists after cerebral ischemia and acute ischemic stroke (Chamorro et al., 2016). It can be seen that inhibiting the activity of NF-κB can reduce the nerve injury after intracerebral hemorrhage or cerebral ischemia (Liu et al., 2019; Shang et al., 2019). In recent years, many studies have reported that the effect of UPS is related to the activation of NF-κB. Intervening UPS can prevent the degradation of IκB and the activity of NF-κB and combine them in cell solute. Ginsenoside Rd treatment can restore IκB expression in the cytoplast after ischemia injury by decreasing proteasome activity, therefore suppressing NF-κB activity and protecting neurons, according to a study (Zhang et al., 2020). Phillips et al. applied proteasome inhibitor PS519 to rats with focal cerebral ischemia and found that PS519 can reduce the inflammatory response by restraining NF-κB and improve the recovery of neurological function in rats with brain injury (Phillips et al., 2000). According to another study, the neuroprotective effect of PS519 may be related to its involvement in the regulation of cell adhesion molecules ICAM-1 and E-selectin, because these two adhesion molecules play a key role in the adhesion and exudation of neutrophils and macrophages across the blood-brain barrier (Berti et al., 2003). These suggest that the effect of proteasome inhibitors on inflammatory response may be multi-channel. Moreover, although early intervention of UPS has a neuroprotective effect on inhibiting NF-κB overexpression at the transcriptional level, the decrease of long-term proteasome activity is related to intracellular protein aggregation, delayed neuronal degeneration, and death (Meller, 2009). It suggested that UPS may played a dual role in neurons after ischemia. Although proteasome inhibitors have been shown to provide protection in cerebral ischemia, other nerve injury problems caused by decreased proteasome activity cannot be ignored (Caldeira et al., 2014). Due to the non-selectivity of most proteasome inhibitors, the application of proteasome inhibitors is limited (Wojcik and Di Napoli, 2004). Chen et al. suggested that selective immunoproteasome inhibitors may be a promising strategy for stroke treatment. They discovered that inhibiting the expression of p65 and reducing infarction volumes in rats may be accomplished by inhibiting the low molecular weight protein 2 (LMP2), a significant catalytic subunit of immunoproteasome (Chen et al., 2015).

### PINK1/Parkin and Cerebral Stroke Regulated by UPS

PINK1/Parkin pathway is one of the classical pathways of mitochondrial autophagy. Under normal conditions, UPS forms a strict PINK1 and Parkin regulatory mechanism to maintain the balance of mitochondrial autophagy and prevent mitochondrial damage caused by excessive mitochondrial autophagy (Oshima et al., 2021). In the stroke induced by mitochondrial dysfunction (Youn et al., 2021; Zhou et al., 2021), PINK1/Parkin pathway mediates ubiquitination of dysfunctional mitochondrial OMM protein, and then clears abnormal mitochondria through crosstalk with autophagy (Geisler et al., 2010). In recent years, there has been more and more studies on this mechanism, which to some extent shows that UPS plays an important role in PINK1/Parkin pathway in the process of brain injury in stroke. At present, the researches on the pathway in stroke mainly focus on its relationship with autophagy, but ignore the role of UPS. In fact, mitochondrial ubiquitination mediated by PINK1/Parkin pathway is the basis of mitochondrial autophagy. Inactive PINK1 cannot activate or recruit Parkin to mitochondria (Narendra et al., 2008; Matsuda et al., 2010). The UPS mechanism can be inhibited to prevent PINK1/Parkin-mediated ubiquitination of OMM proteins (Chan et al., 2011). Furthermore, inhibiting Parkin mitochondrial translocation, lowering Parkin phosphorylation, and lowering the quantity of phosphorylated ubiquitin (pser65 Ub) can all be used to prevent mitochondrial autophagy triggered by various mitochondrial damage causes (Wang et al., 2018a). OMM proteins could be destroyed through an autophagy-independent UPS route, according to Rakovic et al. The bigger OMM proteins MFN2 and TOM70 were only partially ubiquitinated and primarily destroyed by the lysosomal system, whereas the smaller OMM proteins TOM40 and TOM20 were only slightly ubiquitinated and mostly degraded by UPS (Rakovic et al., 2019). It showed that depolarized mitochondrial membrane proteins could be degraded by two different mechanisms: UPS or lysosomal mediated protein hydrolysis. At the same time, the degradation of larger OMM protein affected the stability of OMM, led to its rupture, exposed mitochondrial inner membrane (IMM) to the cytoplasmic environment, and caused drastic changes in IMM structure and morphology, which might eventually lead to the secondary degradation of IMM and matrix protein (Yoshii et al., 2011). In the oxygen-glucose deprivation (OGD) neuronal model, PINK1 knockout mice (PINK1^–/–^) were more sensitive to ischemic injury than control group (Imbriani et al., 2020). Moreover, in traumatic brain injury (TBI), the loss of Parkin would increase the production of ROS, promote oxidative stress and further lead to neuronal death (Mukhida et al., 2005). In a rat model of ischemia, PINK1/Parkin-mediated mitochondrial autophagy may perform a neuroprotective role in hippocampus neurons (Wu et al., 2018). The deletion of either the PINK1 or Parkin genes has been shown to cause aggregated neuronal damage. Increasing the expression of PINK1 and Parkin, on the other hand, could prevent a huge number of nerve cells from dying. Therefore, it is necessary to pay attention to the role of PINK1/Parkin/UPS mechanism in mitochondrial damage after stroke, rather than autophagy.

### UPS Control in Stroke and Keap1-Nrf2

Oxidative stress refers to the imbalance of redox balance caused by the production of excessive ROS after the body is stimulated by the outside world, resulting in the damage of cell tissue (Hybertson et al., 2011). Because of its high oxygen consumption and fat content, the brain is prone to oxidative injury. The role of oxidative stress in ischemia-reperfusion brain injury has long been recognized (Chamorro et al., 2016). Nrf2 is the main regulator of endogenous and exogenous stress defense mechanisms in cells and tissues. Its primary role is to activate the antioxidant response and trigger the transcription of a number of genes in order to protect cells from oxidative stress and restore intracellular homeostasis (Villeneuve et al., 2010; Chen and Maltagliati, 2018). That Nrf2 escape from Keap1 repression is the crucial event for Nrf2-mediated activation (Kaspar et al., 2009). Oxidative stress caused by cerebral ischemia or intracerebral hemorrhage affects the conformation of Keap1. Then, Nrf2 dissociates and transfers to the nucleus, binds to ARE, and stimulates the target gene expression of downstream antioxidant enzymes and other cytoprotective proteins (Hu et al., 2016; Jiang et al., 2017). The expression of Nrf2 is highly up-regulated in ischemic brain tissue, according to studies, and a range of Nrf2 inducers exhibit neuroprotective effects following cerebral ischemia (Wang et al., 2018b). Monomethylfumarate, the immediate metabolite of dimethylfumarate (DMF), causes direct alteration of the cysteine 151 of Keap1, which increases the dissociation of Nrf2 and has neuroprotective properties (Linker et al., 2011; Bomprezzi, 2015). Furthermore, in the human neuroblastoma cell line SH-SY5Y, miR-7 can target to limit Keap1 mRNA translation, preventing the degradation of Nrf2 protein, resulting in Nrf2 activation and cytoprotection (Kabaria et al., 2015). Although these reagents need to be further studied in the stroke model, it is undeniable that preventing Nrf2 from being degraded by proteasome through modification or inhibition of Keap1 is a promising measure in the protection of neurons after stroke. According to a report, p62 expression reduced Nrf2 degradation and increased subsequent Nrf2 nuclear accumulation by inactivating Keap1 (Sun et al., 2016). Li et al. found that sestrin2 stimulated angiogenesis to ameliorate brain injury by activating Nrf2 and modulating the interaction between p62 and Keap1, following photothrombotic stroke in rats (Li et al., 2020). In addition, it has been reported that PINK1/Parkin pathway can promote the release of Nrf2 by inhibiting Keap1 (Xiao et al., 2017). Zhang et al. found that mitophagy reduced oxidative stress via Keap1/Nrf2/PHB2 (Prohibitin 2) pathway after subarachnoid hemorrhage in rats (Zhang et al., 2019). A study has shown that Britanin leading to the induction of the Nrf2 pathway ameliorated cerebral ischemia–reperfusion injury by selectively binding to cysteine 151 of Keap1 and inhibiting Keap1-mediated ubiquitylation of Nrf2 (Wu et al., 2017). Obviously, preventing the proteasome degradation of Nrf2 through the regulation of Keap1 is very important for neuroprotection after stroke.

### HIF-1 Pathway and Stroke Regulated by UPS

Tissue oxygen content plays crucial roles in maintaining the normal functioning of cells and regulation of their development (Mohyeldin et al., 2010). HIF-1 is an important transcriptional regulator of hypoxia and plays an important role in cerebrovascular diseases (Correia and Moreira, 2010). Accumulating evidence indicates that the induction of HIF-1 provides protection against cerebral ischemic damage (Zhang et al., 2011). HIF-1α is widely expressed in the hypoxic/ischemic brain (Chavez et al., 2000). Mice with neuron-specific reduction of HIF-1α that were subjected to temporary focal cerebral ischemia showed higher tissue damage and a lower survival rate, indicating that HIF-1-mediated responses have an overall positive impact in the ischemic brain (Baranova et al., 2007). HIF-1α has complex effects on the brain, which largely depends on the time-point after hypoxic damage. At the earliest post-ischemic stage (i.e., within 24 h), HIF-1α accumulation promotes cell death. In contrast, during the later stage (i.e., >4 days), HIF-1α signaling has a pro-survival effect through limitation of the infarct size (Mitroshina et al., 2021). HIF-1α which is an important regulator of hypoxia being regulated by proteasomal degradation (Shi, 2009). There is now overwhelming data suggesting that the UPS contributes to cerebral ischemic injury and proteasome inhibition is a potential treatment option for stroke (Wojcik and Di Napoli, 2004). Growing evidence shows that proteasome inhibitors enhance angioneurogenesis and induces a long-term neuroprotection after cerebral ischemia. Inhibition of immunoproteasome LMP2 was able to enhance angiogenesis and facilitate neurological functional recovery in rats after focal cerebral ischemia/reperfusion. A study highlights an important role for inhibition of LMP2 in promoting angiogenesis events in ischemic stroke, and point to HIF-1α as a key mediator of this response (Chen et al., 2018). The novel proteasome inhibitor BSc2118 protects against cerebral ischemia through HIF1A accumulation and enhanced angioneurogenesis (Doeppner et al., 2012). Although the role of HIF-1α in cerebral ischemia remains complex, the role of HIF-1α as mediator of BSc2118-induced neuroprotection is appealing based on the data present (Yan et al., 2011). Furthermore, a result indicates that 20S proteasomes are involved in HIF-1α degradation in ischemic neurons and that proteasomal inhibition provides more HIF-1α stabilization and neuroprotection than PHD inhibition in cerebral ischemia (Badawi and Shi, 2017).

## Conclusion and Future Directions

UPS is the main pathway for the degradation of cytosolic, nuclear and transmembrane proteins, and also the main regulator for maintaining neural development, brain structure and function (Park et al., 2020). Neuron is a highly differentiated terminal cell. Various components of UPS widely exist in synapses and participate in the regulation of synaptic function (Tsakiri and Trougakos, 2015). After stroke, due to the destruction of the internal environment of neuron survival, a series of neuron injury events are caused, which eventually leads to the death of nerve cells and the loss of nerve function. In recent years, there have been more and more researches on UPS. It is found that UPS mediated protein degradation is an important mechanism for the body to regulate the level and function of intracellular proteins. The components involved in this biological process mainly include ubiquitin and its related starting enzymes. UPS plays a very important role in maintaining cell homeostasis (Shang and Taylor, 2011). At the same time, UPS is also involved in the pathological process related to nerve injury after stroke (Ge et al., 2007). At present, there are many research results on the physiological and pathological mechanism of stroke. However, the discussion on UPS and stroke is insufficient and there is a lack of literature for reference. Nevertheless, it may still become a new hotspot in basic research and potential clinical application. It should be noted that the aggregation of ubiquitinated proteins is one of the important features after stroke (Luo et al., 2013). UPS is closely related to the pathways of post-stroke related pathological changes such as mitochondrial autophagy, oxidative stress, hypoxia and inflammatory response (Ahmad et al., 2014). To study the role of UPS in PINK1/Parkin, NF-κB, HIF-1α and the regulatory mechanism of Keap1-Nrf2 pathway is of great significance for the clinical treatment and prognosis of stroke patients. At present, there are still many problems that need more experiments to study. From the perspective of maintaining homeostasis, it is necessary to clarify how to moderately activate or inhibit UPS to play a cytoprotective role. The pathological process of stroke is a dynamic process. It is necessary to observe the changes of UPS by stages. Due to the lack of research on the side effects of drugs in experimental animals and the results of clinical trials, the conclusion whether UPS regulating drugs mediate cell protection or cytotoxicity after stroke is still controversial. To explore the relationship between UPS and stroke and its mechanism has great potential to improve the prognosis of stroke patients.

## Author Contributions

Y-CL wrote the manuscript. WZ and YW proofread the manuscript. All authors read and approved the final manuscript.

## Conflict of Interest

The authors declare that the research was conducted in the absence of any commercial or financial relationships that could be construed as a potential conflict of interest.

## Publisher’s Note

All claims expressed in this article are solely those of the authors and do not necessarily represent those of their affiliated organizations, or those of the publisher, the editors and the reviewers. Any product that may be evaluated in this article, or claim that may be made by its manufacturer, is not guaranteed or endorsed by the publisher.
